# Versatile Vibrational Energy Sensors for Proteins

**DOI:** 10.1002/anie.202200648

**Published:** 2022-04-06

**Authors:** Jan G. Löffler, Erhan Deniz, Carolin Feid, Valentin G. Franz, Jens Bredenbeck

**Affiliations:** ^1^ Institute of Biophysics Goethe University Frankfurt Max-von-Laue-Straße 1 60438 Frankfurt (Main) Germany

**Keywords:** Non-Canonical Amino Acids, Protein Dynamics, Protein Modifications, Time-Resolved Spectroscopy, Vibrational Energy Transfer

## Abstract

Vibrational energy transfer (VET) is emerging as key mechanism for protein functions, possibly playing an important role for energy dissipation, allosteric regulation, and enzyme catalysis. A deep understanding of VET is required to elucidate its role in such processes. Ultrafast VIS‐pump/IR‐probe spectroscopy can detect pathways of VET in proteins. However, the requirement of having a VET donor and a VET sensor installed simultaneously limits the possible target proteins and sites; to increase their number we compare six IR labels regarding their utility as VET sensors. We compare these labels in terms of their FTIR, and VET signature in VET donor‐sensor dipeptides in different solvents. Furthermore, we incorporated four of these labels in PDZ3 to assess their capabilities in more complex systems. Our results show that different IR labels can be used interchangeably, allowing for free choice of the right label depending on the system under investigation and the methods available.

## Graphical abstract

A tool kit of non‐canonical amino acids for site‐resolved sensing of vibrational energy transfer (VET) in proteins is established. The actual sensor is an azide or nitrile group attached to different side chains for compatibility with different protein sites. Ultrafast spectroscopy experiments on peptides and a protein ensure comparability of the sensors, allowing for choosing the best for a given site.

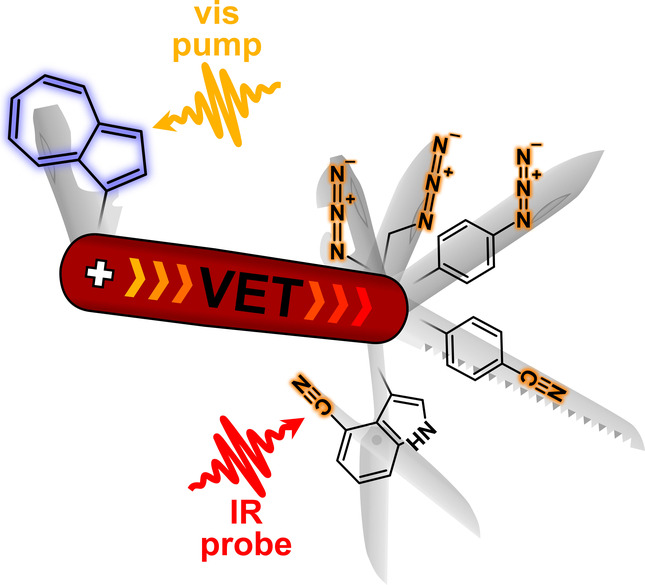

## Introduction

Non‐canonical amino acids (ncAAs) are being used to extend the functions and properties of proteins and peptides beyond the scope of their natural counterparts. Such ncAAs containing suitable side chains have been used for example as spectroscopic labels, as photo‐crosslinkers, for introduction of heavy atoms, as artificial redox centers or as photoswitchable modulators of protein conformation.^[1]^ Depending on the ncAA, different incorporation strategies are available, including solid‐phase peptide synthesis (SPPS), chemical modifications (e.g., using click‐chemistry), selective pressure incorporation (SPI), and stop codon suppression (SCS), each of which have their own drawbacks and advantages.^[2]^

**Figure 1:**
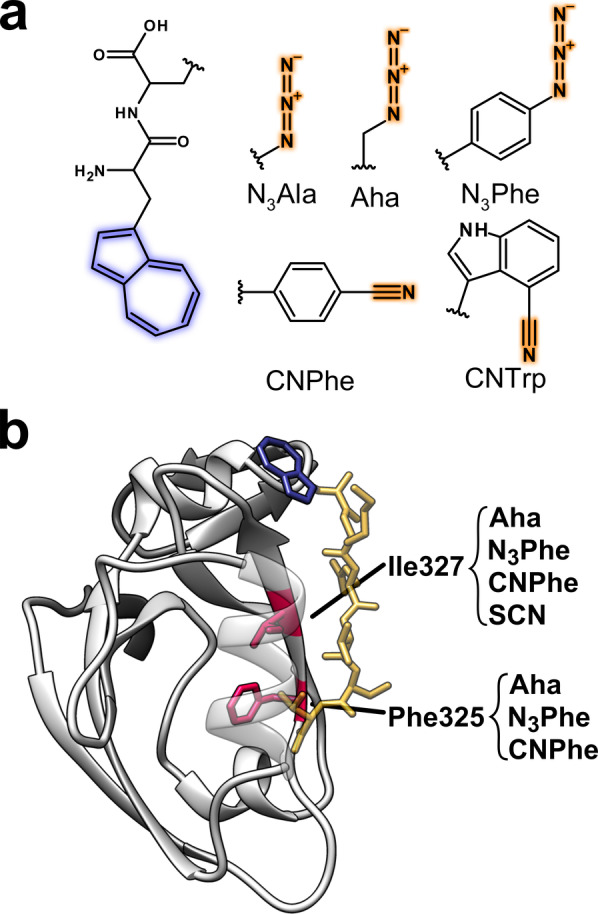
a) Chemical structures of the dipeptide scaffold and the different vibrational probes. Each dipeptide consists of the AzAla moiety (highlighted in blue) for VIS excitation and one of the ncAAs for IR probing (probed atom groups are highlighted in orange). b) Schematic of the PDZ structure (gray) with the ligand (yellow) containing AzAla (blue). The indicated residues (purple) have each individually been replaced by Aha, N_3_Phe and CNPhe, Ile327 was additionally replaced with SCN labeled cysteine (structure based on pdb: 1BE9^[12]^).

**Figure 2:**
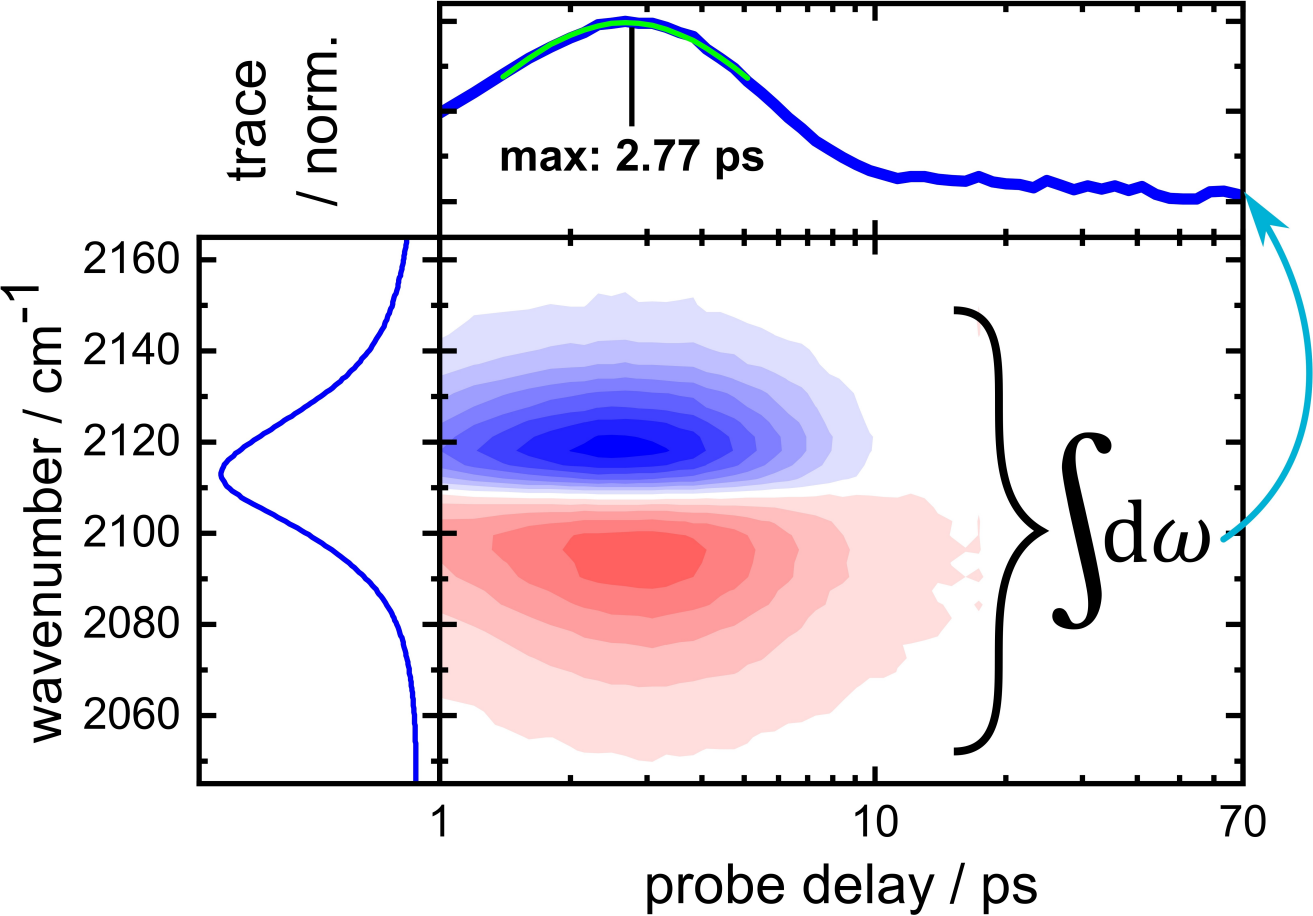
Example of a TRIR dataset of AzAla–Aha in H_2_O. (left) FTIR spectrum of the azide mode in the wavelength region probed in the TRIR experiment. Bottom right: A typical VET signature in the TRIR difference spectrum, consisting of decreased (blue) and increased (red) absorbance, caused by a redshift of the absorption band upon arrival of vibrational energy at the low‐frequency modes that are anharmonically coupled to the probed mode. Top: Normalized trace of the TRIR spectrum, calculated by integrating over the absolute signals of the TRIR spectrum at each delay. A biexponential fit (green) of the upper 35 % of the trace is used to determine the peak time, 2.77 ps in this data set.

Previously, we established a set of two ncAAs that can be used to experimentally track vibrational energy transfer (VET) in proteins or peptides in a site‐specific manner.[^3^, ^4^, ^5^] In ultrafast VIS‐pump/IR‐probe (TRIR) experiments the enzymatically available^[6]^ β‐(1‐azulenyl)‐alanine (AzAla; Figure 1a)^[7]^ can be used to locally introduce vibrational energy into a peptide system. Exciting the azulene moiety with a 600 nm photon results in an excited S_1_ state that decays on a sub‐ps timescale via internal conversion. The fast relaxation is crucial to generate a well‐defined onset of the VET. The internal conversion leads to the population of low‐frequency modes in the vicinity of AzAla. Because they are less localized than high‐frequency modes,^[8]^ their couplings allow for the energy to spread through the system.[^9^, ^10^, ^11^] Azidohomoalanine (Aha) is used to detect the energy at another distinct location in the peptide, as the wavenumber of the azido's asymmetric stretching vibration undergoes a shift, due to coupling of the observed vibration to low‐frequency modes that receive the vibrational energy (Figure 2).[^3^, ^10^, ^13^] Consequently, the VET signal decays together with the population of those low‐frequency modes, leading to peaking of the signal on a ps timescale. This is in stark contrast to the signals that may arise due to the sample heating. On the timescale of our experiments such effects occur late, are small compared to the VET signal for the vibrational probes and remain constant once the equilibrium is established. Noticeable heating signals are only observed for the strong water background absorption in aqueous samples which are subtracted as shown in Figure S1. The thermal signature of DMSO and THF is negligible.

Such VET experiments are of growing interest, as VET appears to play a crucial role for various protein functions. It has been shown that pathways of allosteric connectivity overlap with pathways of efficient energy transfer.^[14]^ VET has been suggested to play a key role in the dissipation of excess reaction energy in catalytic and photoreactive proteins.^[15]^ The idea of underdamped collective protein motions that influence enzymatic turnover through coupling to the reaction coordinate (rate promoting vibrations) is gaining an increasing amount of interest.^[16]^ Recent findings demonstrate that protein residues participating in such motions coincide with efficient energy transfer pathways.^[17]^

While the incorporation of the two VET labels via SPPS of small peptides is straightforward, the applicability to full‐sized proteins is limited. Cotranslational AzAla incorporation has been achieved using SPI in *L. lactis*,^[18]^ and recently an aminoacyl‐tRNA synthetase/tRNA pair was developed to incorporate AzAla via SCS in *E. coli*.^[4]^ However, the VET sensor Aha can so far only be incorporated into proteins via SPI as a replacement of methionine, limiting flexible positioning to Met‐free proteins only.^[19]^

To increase the applicability and extend the field of potential systems to investigate in ultrafast VET experiments, it is necessary to introduce additional VET donors and sensors that can be selected according to the individual requirements of each protein system. Ultimately, one would like to be able to choose between different ncAAs for positions that strictly require aromatic or aliphatic substitutions, or where steric reasons prohibit or require a certain size of the amino acid.

Azides and nitriles are commonly used as IR labels in proteins with both labels being readily incorporated as ncAAs.[^19^, ^20^] The large extinction coefficient *ϵ* of the azide moiety makes it an attractive IR sensor for linear and, in particular, non‐linear spectroscopy, because the signal scales with *ϵ* ^2^. However, the frequent occurrence of Fermi resonances[^21^, ^22^, ^23^] can distort band shapes. The *ϵ* of the CN‐stretching vibration is lower by a factor of up to 4, and that of the thiocyanate (SCN) label is typically even lower.[^24^, ^25^]

There are various aspects that determine the amplitude and dynamics of a VET signal. The *ϵ* of the IR probe linearly affects the VET signal amplitude. The anharmonic coupling to low‐frequency modes leads to the shift of the probed mode, and hence the amplitude of the induced difference signal.^[10]^ The band width of the probed mode needs to be considered, as a VET‐induced shift by a certain amount will lead to a stronger signal for a narrower band. The size of the employed probe will change the final amplitude and dynamics of the VET signal, due to the number of its internal degrees of freedom. Such effects of the probe are expected to be more important in small systems, whereas they are negligible in larger proteins. The vibrational lifetime of the probe does not directly influence the VET measurements, but a particularly long lifetime may hint at weak anharmonic couplings, as such couplings are one factor determining a probe's lifetime. Due to the complexity of these simultaneous influences, an experimental comparison of different VET labels is required to design successful VET studies in the future.

Here, we compare the potential VET sensors azidoalanine (N_3_Ala), Aha, 4‐azido‐phenylalanine (N_3_Phe), 4‐cyano‐phenylalanine (CNPhe), and enzymatically synthesized^[26]^ 4‐cyano‐tryptophan (CNTrp) in dipeptides containing AzAla as VET donor (Figure 1a). Furthermore, VET to Aha, N_3_Phe, and CNPhe at two distinct sites in a protein–ligand complex (Figure 1b) and cyanylated cysteine (S^13^C^15^N abbreviated as SCN) at one of these sites was analyzed. With this assessment of the VET signals in various environments (different solvents and within a protein) a comprehensive comparison of the IR VET sensors is achieved.

## Results and Discussion

VET‐induced band shifts observed in VIS‐pump/IR‐probe or 2D‐IR experiments are typically small compared to the band width.[^5^, ^10^] They are determined by the anharmonic couplings of the probed vibration to the low frequency modes populated by VET. However, the experimental measurement of the coupling constants is difficult. Another important factor for the size of the VET signal is the band width of the probe: Due to cancelation between bleach and induced absorption, the VET signal is, in the case of a given small shift, inversely proportional to the full width at half maximum (FWHM) of the reporter mode (Figure S2). As the band widths differ by up to a factor 5 between the probes, this has to be taken into account when comparing them. Furthermore, the VET signal will be proportional to the extinction coefficient of the label. The spectral position of the label is another aspect to consider, though this property does not influence the signal size in the VET experiments. All IR labels investigated in this study have their characteristic absorption in a transparent spectral window of aqueous protein samples.^[27]^ But even in this region a distinct H_2_O combination band determines the experimental conditions. The azide band lies on top of this solvent band and the nitrile band on its slope. In D_2_O both bands coincide with the slope of the O–D stretching vibration,^[28]^ but the optical density is larger in the region of the nitrile band. These bands will therefore affect the maximum path length, transmitted light, signal‐to‐noise ratio, and will lead to different background signal strengths and shapes. Before turning to the VET experiments, we therefore investigated the labels in their different environments by FTIR spectroscopy.

In the following, the results of the FTIR experiments are presented for the dipeptides first, followed by the proteins, before turning to the discussion of the VET experiments, in the same order.

**Figure 3:**
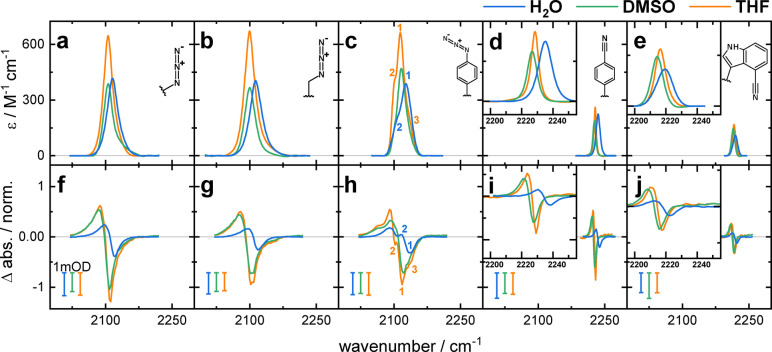
a)–e) Extinction coefficients of the IR band of the dipeptides containing N_3_Ala, Aha, N_3_Phe, CNPhe, and CNTrp, respectively (indicated by the structures). f)–j) Difference spectra of the VET experiments at the time of maximum signal intensity of the dipeptides containing the same IR labels as above. The signal intensity is scaled to the peptide concentration of each experiment, so that signal amplitudes can be compared. Colored bars indicate 1 mOD difference signal in the VET experiments. Each panel contains the spectra of the respective dipeptide in H_2_O (blue), DMSO (green), and THF (orange). The spectral range covered in all panels is identical, to better visualize the difference in band width of the different IR probes, the insets show the narrow nitrile band shape in detail. Small numbers in the FTIR and VET spectra of AzAla–N_3_Phe indicate the presence of multiple subbands.

The FTIR spectra of the dipeptides (Figure 3a–e) show distinct absorption bands of the probes with peaks between 2100 cm^−1^ and 2126 cm^−1^ for the azides, and 2214 cm^−1^ to 2235 cm^−1^ for the nitriles (for a complete list of the FTIR parameters, see Supporting Information Table S1). As mentioned before, we expect a stronger VET signal for narrow bands. All probes show a hypsochromic shift in H_2_O compared to the other solvents. Additionally, the absorption band is broadest in H_2_O. This effect is especially pronounced in the aromatic probes: the band width of the CNPhe peptide increases by 80 % in H_2_O as compared to THF. In order to maximize the VET signal amplitude, it is therefore advisable to place the VET probe in the hydrophobic protein interior instead of a solvent‐exposed site. The extinction coefficient *ϵ*_max_ at the band maximum of the nitriles is ≈4 times lower than that of the azides, at the same time their FWHM is up to 5 times narrower. This leads to a 5–16 times lower integrated extinction coefficient of the nitriles vs. the azides.

The variation in the extinction coefficients of the different probes, depending on the solvent, and, in labeled proteins, on the environment of the label, is in good agreement with the literature.^[29]^ The wavenumber shift of the azide absorption is dominated by the H‐bond donor strength of its surroundings,[^30^, ^31^] as evidenced by the strong blueshift in H_2_O compared to the aprotic solvents DMSO and THF, which feature different polarities and permittivities but do not cause strong shifts. For the nitrile band we observe an increasing red shift from H_2_O, to THF and DMSO (CNPhe: 2235 cm^−1^, 2228.4 cm^−1^, 2226.7 cm^−1^; CNTrp: 2219.3 cm^−1^, 2216.4 cm^−1^, 2214.4 cm^−1^), in accordance with previous studies.[^25^, ^30^, ^32^]

While the nitriles have a single, symmetric band, the azides show up to two additional subbands. In N_3_Phe these appear as shoulders on the high‐ and low‐wavenumber slope of the band. This three‐band structure has been attributed to Fermi resonances between the azide asymmetric stretching vibration and lower wavenumber combination bands.^[23]^

For N_3_Ala and Aha such subcontributions are less pronounced: the aliphatic azides only show a slight asymmetry at the blue side. However, this asymmetry has also been identified as a Fermi resonance in N_3_Ala using 2D‐IR spectroscopy.^[21]^

As we analyze integrated signals, probes exhibiting Fermi resonances are not generally inappropriate for VET application and the additional coupling might even lead to a stronger response. However, the hardly predictable band shape may lead to lower or higher signal amplitudes than expected from the isolated ncAA, as discussed below.

**Figure 4:**
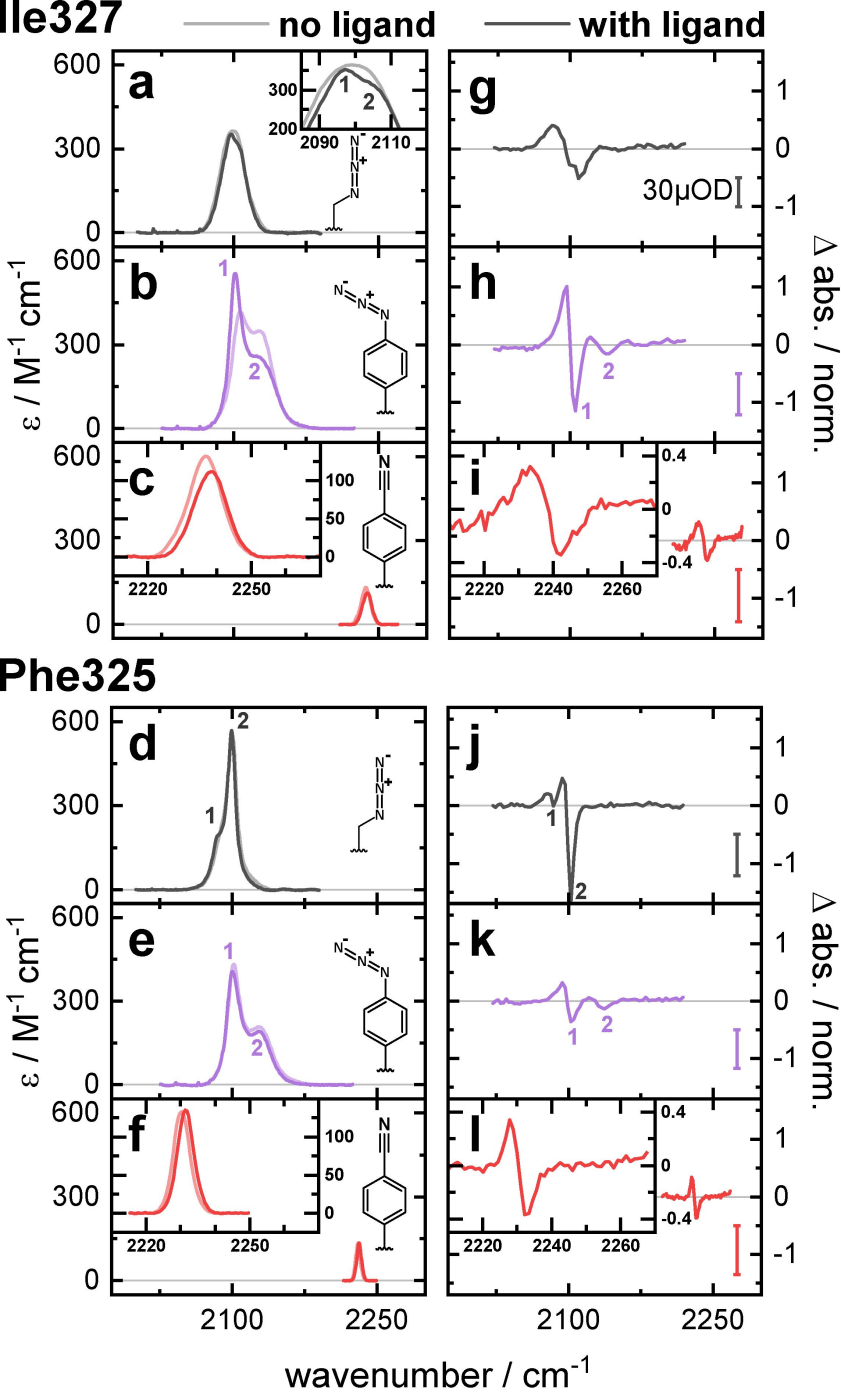
a)–f) Extinction coefficients of PDZ variants with Aha (gray), N_3_Phe (purple), and CNPhe (red), as indicated by the structures. Each panel shows the extinction coefficient without the ligand (dim colors) and with the ligand (intense colors). g)–l) Split sample cell^[33]^ difference spectra of the VET experiments at the time of maximum signal intensity of the PDZ mutants (same colors as in the left panels). The upper panels show the results of PDZ with labels inserted at Ile327, the lower panels show data for PDZ labels inserted at Phe325 instead. The VET signal intensity is scaled to the protein concentration and to the difference signal of the water at 2212 cm^−1^ for each experiment leading to comparable signal amplitudes. Colored bars indicate 30 μOD difference signal in the VET experiments. Small numbers in the FTIR and VET spectra of Aha and N_3_Phe containing mutants indicate the presence of multiple subbands.

The extinction coefficients of the labels Aha, N_3_Phe and CNPhe inserted into the protein PDZ are in the same range for both labeling sites as those obtained for the dipeptides (Figure 4; for an enlarged view including the results of the dipeptides see Figure S3). The absorption bands of Aha and N_3_Phe at both sites contain at least two contributions.

The absorption of Ile327Aha consists of two subcontributions, that become evident upon binding of the AzAla–KQTSV ligand, which leads to a slightly reduced relative intensity in the high‐wavenumber component. Phe325Aha, on the other hand, is distinctively different from the band shape of the corresponding dipeptide in either solvent. A narrow and intense band around 2100 cm^−1^ is complemented by a low‐wavenumber component at 2088 cm^−1^, upon ligand binding this low‐wavenumber component shifts by −2 cm^−1^, leading to the formation of a clear shoulder. However, the main feature remains practically unperturbed by the binding event. A certain interpretation of this shift is not possible, especially since this sensitive subcontribution is likely caused by Fermi resonances.

The N_3_Phe label at both sites within the protein leads to a band shape very different from those of the AzAla–N_3_Phe dipeptide in different solvents. An intense and narrow peak is accompanied by a high‐wavenumber contribution. At Ile327N_3_Phe, the ligand binding leads to a narrowing of the low‐wavenumber component together with broadening of the high‐wavenumber component (Figure S4). This leads to a relative enhancement in the amplitude of the low‐wavenumber component. A redshift of both components may indicate reduced polarity, but it is pointed out, that the solvatochromic response of the N_3_Phe moiety is somewhat ambiguous.^[30]^ At Phe325N_3_Phe the binding of the ligand has no effect on the overall band shape in accordance with the indications of the Aha label.

The FTIR absorption band of CNPhe consists of a single band at both protein sites. The FWHM of 11 cm^−1^ in Ile327CNPhe is comparable to that of the AzAla–CNPhe dipeptide in H_2_O, despite being in the protein interior, Phe325CNPhe has a FWHM of 5 cm^−1^ similar to the dipeptide in THF.

The peak position of Ile327CNPhe at 2237 cm^−1^ indicates that the amino acid experiences a less polar environment than in Phe325CNPhe (2230 cm^−1^), upon ligand binding both bands shift about 1 cm^−1^ to higher wavenumbers, without further changes.

**Figure 5:**
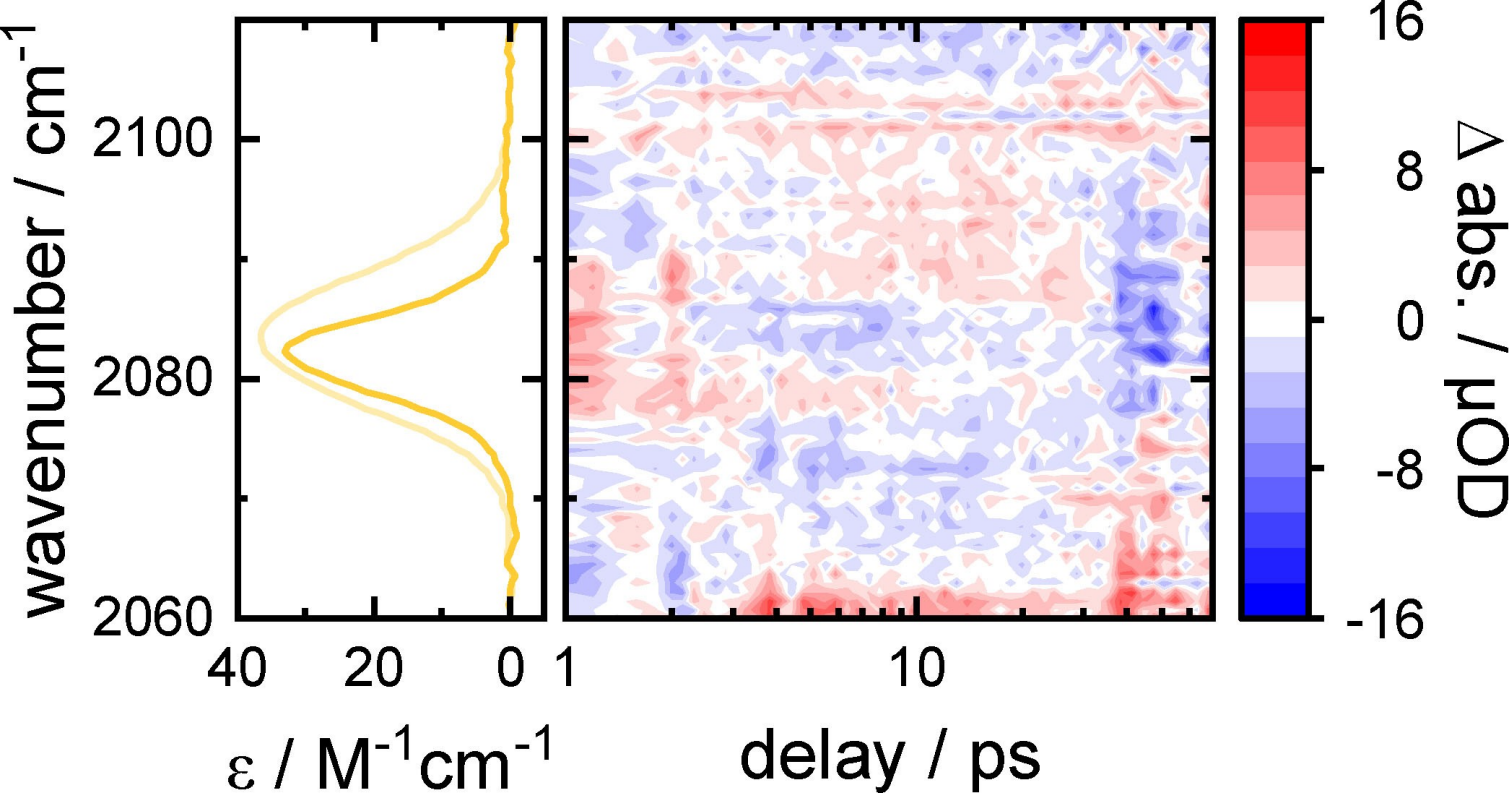
Left: FTIR extinction coefficient of PDZ Ile327SCN without and with ligand in light yellow and yellow, respectively. Right: TRIR data of PDZ Ile327SCN, despite a noise level of a few μOD, no clear VET signature emerges.

The thiocyanate labeled PDZ Ile327SCN (Figure 5) displays a distinct absorption at 2083 cm^−1^ with an extinction coefficient of 36 M^−1^ cm^−1^, similar to previously reported findings.^[25]^ Binding of the ligand leads to a decrease in the band width of the label.

All five dipeptide samples show an intense VET signature in the TRIR experiments in all three solvents (Figure 3f–j). The spectra are scaled to compensate for different concentrations, so relative amplitudes can be compared between different labels and solvents. The scale bars allow to read off the unscaled signal sizes. The spectral position and the substructure of the VET signatures is in accordance with the FTIR spectra. For all dipeptides, we observe the lowest VET signal amplitude in H_2_O and the signal in DMSO and THF is stronger by a factor of 2–4.

Besides the solvent, the band shape also influences the signal amplitudes. As mentioned before, the difference signal is induced by a shift and possibly slight widening of the absorption due to the deformation of the anharmonic potential of the reporter mode. In cases with a band shape consisting of multiple features, this may lead to indentations within the bleach or the induced absorption (as seen in N_3_Phe) and an overall decrease in amplitude due to cancelation effects.

**Figure 6:**
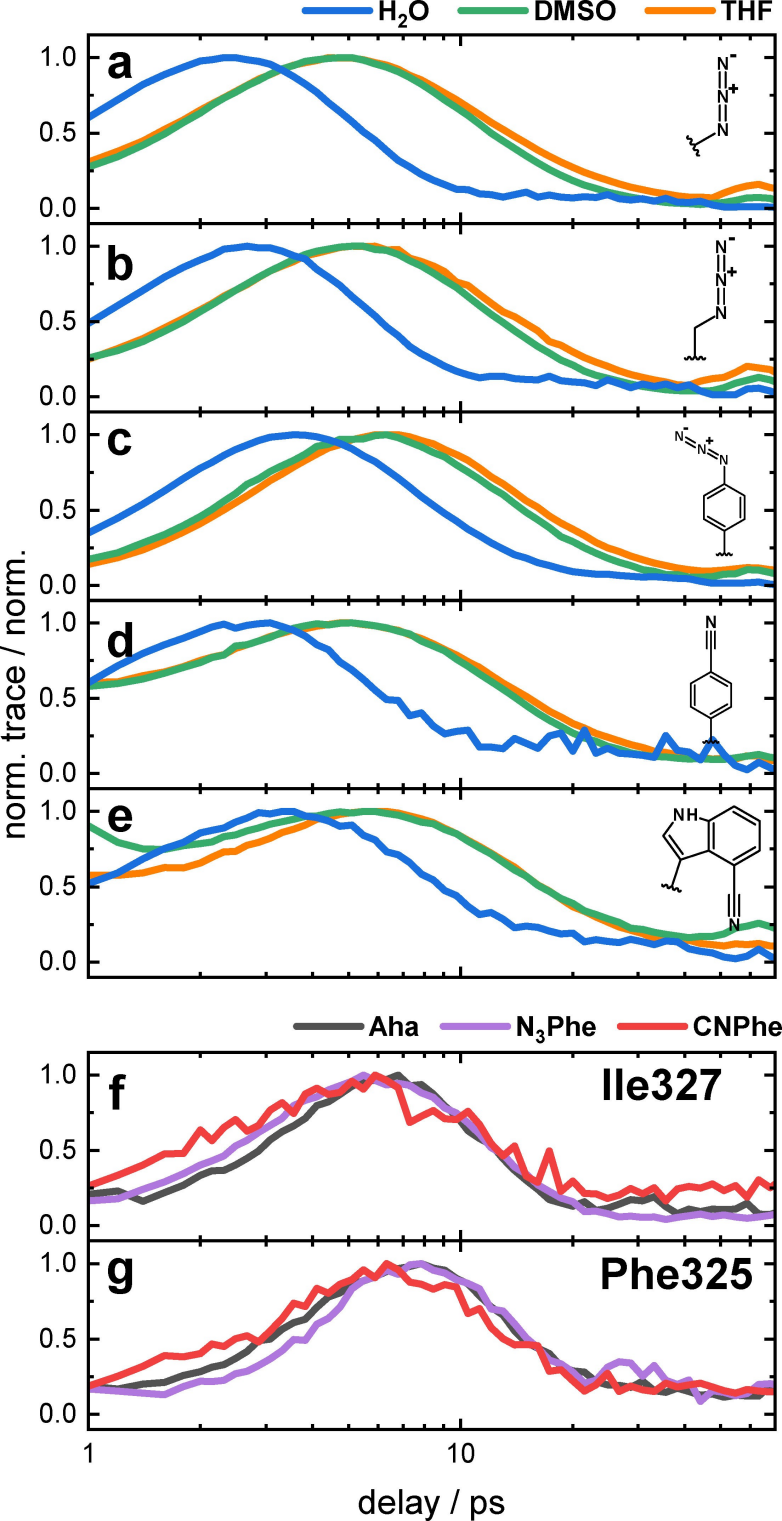
a)–e) Normalized VET traces of the dipeptides in H_2_O (blue), DMSO (green), and THF (orange). The side chain structure of the respective IR probe is shown within the panels. f), g) Normalized VET traces of the PDZ variants with Aha (gray), N_3_Phe (purple), and CNPhe (red) at the positions indicated within the panels.

**Figure 7:**
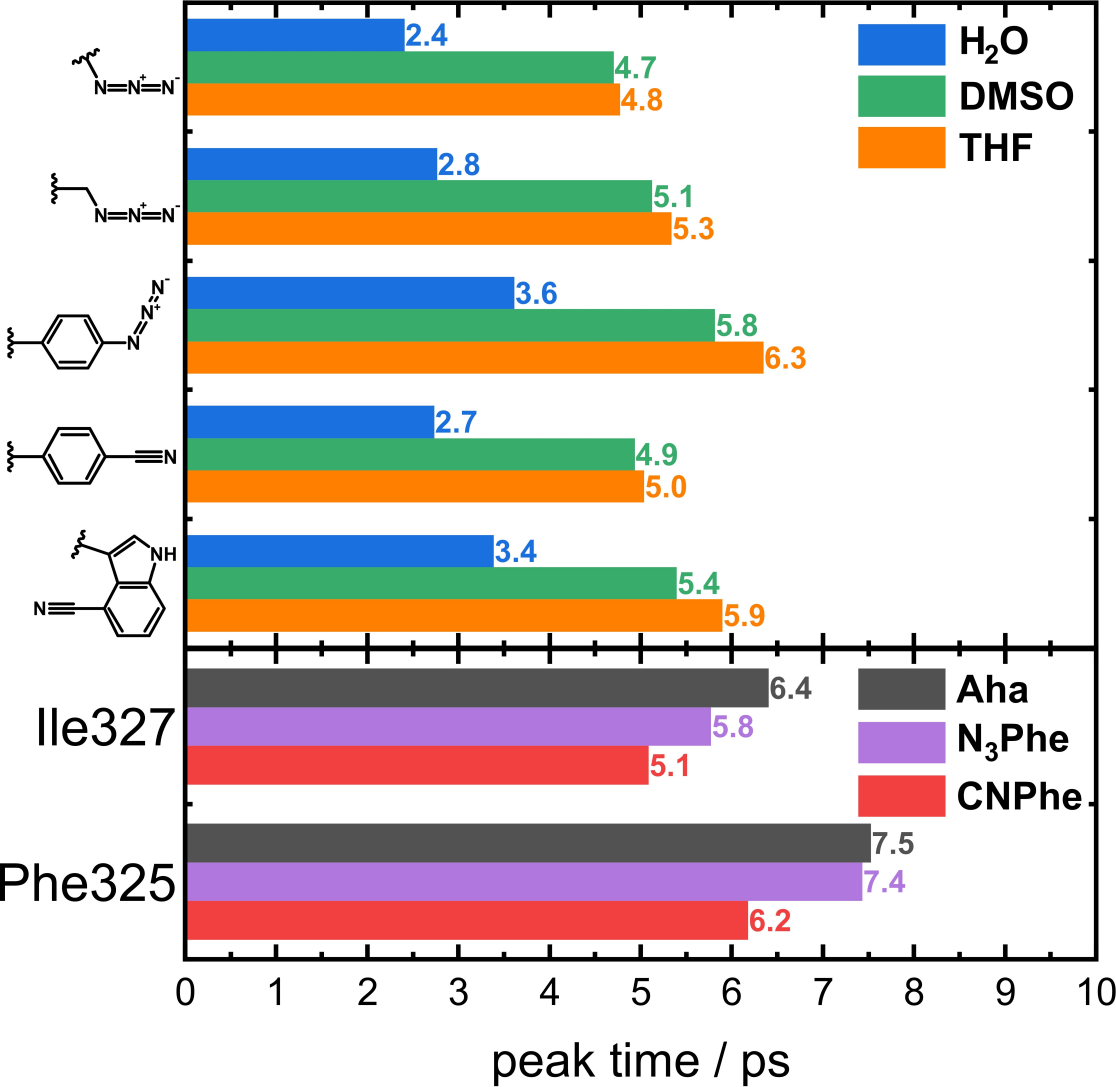
Top: Bar plot of the VET peak times of the dipeptides in H_2_O (blue), DMSO (green), and THF (orange). The IR probe for the respective group of three bars is indicated at the left. Bottom: Bar plot of the peak times of the PDZ variants with Aha (gray), N_3_Phe (purple), and CNPhe (red) at the sites indicated at the left.

In the different solvents, the peak times of the dipeptides shift in a similar fashion as the signal's amplitude (Figure 6a–e and Figure 7 top). The peak time in H_2_O is the earliest for all dipeptides. In DMSO the signal peak is reached on average 76 % later than in H_2_O, a similar shift of on average 85 % is observed between H_2_O and THF. This behavior observed for all dipeptides likely arises from the fast cooling of the dipeptides in water as compared to DMSO and THF.

Additionally, the size of the label has an influence on the peak time. From N_3_Ala to Aha, the peak time increases on average 12 % and again by 21 % from Aha to N_3_Phe. When the phenyl ring of CNPhe is exchanged for the larger indole ring of CNTrp, the peak time increases by 16 %.

Despite the complexity of the signal formation, the peak time is a good measure for the overall dynamics of the VET,[^5^, ^11^, ^34^] as is also evident from the systematic behavior of the various dipeptides in the different solvents.

The reduction of the peak time between N_3_Phe and CNPhe indicates, how the reporter mode alone influences the dynamics of the VET signal (with a bridging phenyl ring in both peptides). Switching from nitrile to azide, results in a delay of the peak time by 0.9–1.3 ps. From the longer vibrational lifetime of the nitrile compared to the azide (N_3_Ala: 1.1 ps;^[21]^ Aha:1 ps;^[35]^ N_3_Phe: 0.8 ps;^[36]^ CNPhe: 4 ps;^[37]^ CNTrp: 1.4–1.9 ps^[38]^), one might have expected that also the VET signal of the nitrile is reaching its maximum later. The timescales of the decay of the VET signal are also quite similar among the different probes in the same solvent or at the same protein site as can be seen in Figure 6, while the vibrational lifetimes vary by up to a factor 5 between CNPhe and N_3_Phe. However, different lower frequency modes can be important for VET as compared to vibrational relaxation. Therefore, the effect of changing the reporter group needs to be characterized for each potential VET sensor individually and cannot be deduced from vibrational lifetimes.

The VET signal shapes of the protein samples reflect the band shape of the FTIR spectra, as they did in the dipeptides. The azide probes display complex band shapes with multiple overlapping features, whereas the VET signals of CNPhe represent a single bleach and a single induced absorption feature (Figure 4g–l).

The signal intensities of the azide probes vary strongly, depending on the band shape of the probe at the respective site. At Ile327 the N_3_Phe VET signal has the highest amplitude, at Phe325 an extraordinarily pronounced bleach of Aha dominates the signals of the other probes. The signal intensity of Ile327CNPhe is 25 % lower than that of Ile327Aha, and that of Phe325CNPhe is 10 % larger than for Phe325N_3_Phe.

In the PDZ mutants Ile327N_3_Phe and Phe325Aha, the Fermi resonances of the azide bands lead to a strong enhancement of the VET amplitude. In both cases, a narrow and intense band with a side peak emerge. In Ile327N_3_Phe the weaker contribution is blue‐shifted compared to the main feature. In this mutant, the cancelation by the induced absorption on the bleach is less severe, leaving the bleach and the induced absorption of the main feature almost unperturbed. In Phe325Aha, the additional band is red‐shifted compared to the main feature. This leads to cancelation effects in the induced absorption of the main feature, making the narrow bleach 3.6 times stronger than the apparent amplitude of the induced absorption.

Due to the extraordinary band shape of these mutants (Ile327N_3_Phe and Phe325Aha), their intensity does not represent the typical signal amplitude of these IR labels, but rather extreme cases. The CNPhe label on the other hand has a well‐preserved signal shape in all investigated systems, making its signals more predictable. Comparing Ile327Aha and Phe325N_3_Phe with the corresponding CNPhe mutants reveals a 25 % lower and a 10 % higher signal strength, respectively. Not only in the dipeptides, but also in the complex protein system, the CNPhe VET signal is stronger than the extinction coefficient alone would suggest, underlining the additional importance of the narrow band width for the signal amplitude.

Based on the findings of the dipeptides, one would expect the peak times in the protein at each site to be equal for Aha and CNPhe and increased by ≈1 ps for N_3_Phe. Instead, a decrease in the peak time from Aha to N_3_Phe and from N_3_Phe to CNPhe is evident (Figures 6f,g and 7). We attribute this behavior to the protein environment. In the dipeptides, few pathways participate in the VET to the sensor and into the solvent. Within the protein, countless connections between AzAla and the protein contribute to the overall signal, making the response more specific for the IR probe and less susceptible to each individual step in the VET process including the solvent dissipation.

At both protein sites, the energy maximum is reached in the same order depending on the probe: CNPhe peaks first, followed by N_3_Phe, and then Aha.

The peak time increases between Ile327 and Phe325 by 1.1, 1.6, and 1.1 ps for Aha, N_3_Phe, and CNPhe, respectively, nicely reflecting the increased distance between AzAla and the respective labeling site. The peak times of all different probes at each site lie within 1.3 ps, defining a window in which the peak times of varying labels in proteins can be considered the same. These differences can be attributed to differences in the timescale of energy redistribution within the label and differences in the coupling between the probed mode and the low‐frequency modes of the label. The relative impact of these differences will be smaller for longer peak times.

A VET measurement and data treatment of the cyanylated cysteine Ile327SCN variant of PDZ were conducted in the same manner as for all other protein samples. However, no clear VET signature could be observed (Figure 5). It has been shown that heavy atoms that link a vibrational label to the rest of a molecule, can insulate this group from the remaining system, leading to prolonged vibrational lifetimes of these groups as compared to a bond facilitated by carbon.^[39]^ This insulation effect may be the reason for the absence of a VET signature in this PDZ variant. We conclude that the heavy sulfur atom uncouples the nitrile group from the rest of the protein system and thereby prevents the population of low‐frequency modes that could couple to the probed mode.

We propose that the signal size of the nitriles is based on a higher ratio between the resulting shift and the FWHM of the probe, compared to the azides. Due to the narrow band width, a shift of the CN absorption band leads to a more intense difference signal than the same shift on an equally strong azide signal.

When one attempts to fit the VET data, by shifting and subtracting a scaled version of the FTIR signal, one systematically gets too high induced absorption and too little bleach (Figures S6–S9). For the dipeptide datasets with simple band shapes (IR probes: N_3_Ala, Aha, CNPhe, CNTrp) a better fit can be achieved by fitting the FTIR peak with a Voigt function to represent the bleach and a shifted Voigt function with the same area to model the induced absorption. However, both methods (shifted FTIR and Voigt fit) result in almost the same VET‐induced shifts with dynamics nicely reproducing the trace obtained by integration.

Using the approach published before,^[40]^ we can estimate an upper limit for the bleach, which is 12 % of the FTIR signal, if the bleach and induced absorption were fully separated (assuming a Gaussian intensity distribution of the beams, magic angle conditions, and a quantum yield of the internal conversion of 1). With this upper limit for the amplitude, we can determine a lower limit for the shift at the maximum signal.

We observe shifts between 0.78 cm^−1^ and 2.3 cm^−1^ for the two azides and 0.36 cm^−1^ to 0.94 cm^−1^ in the nitriles. In H_2_O the relative shifts (shift/FWHM) of all labels are low (2.5 % for Aha; ≈3.5 % for all other probes). The relative shift of the azides is largest in DMSO (9 % and 8 %) like the shift of CNTrp (8.5 %), whereas CNPhe shifts by almost 13 % relative to its FWHM, and almost the same if measured in THF. This explains why the signal of CNTrp in these solvents is lower than that of CNPhe, in which the reduced *ϵ* is compensated by a stronger relative shift compared to the azides.

## Conclusion

To investigate the nature and various implications of VET for the functions of proteins, TRIR spectroscopy in combination with various artificial amino acids is a valuable tool. However, the previously established label Aha was limited to application in Met‐free proteins and may not be suitable for substitution in all positions within the variable scaffolds of different proteins.

The presented results show that different moieties can be used as labels for VET experiments. Some of the ncAAs can be incorporated via SCS, instead of SPI, greatly increasing the number of target proteins. Furthermore, these labels now include aromatic side chains, allowing the investigation of aromatic protein sites, without altering the chemical characteristic of the exchanged residue, and they are of various sizes, allowing to choose the right ncAA to substitute larger or smaller residues.

We have demonstrated that nitrile and azide moieties can be used for VET experiments with less signal reduction than expected from the *ϵ* alone. The effects of a lower extinction coefficient of the nitrile moiety are largely compensated by its narrow band width. On the other hand, the Fermi resonances of the azide moiety result in less predictable signal amplitudes. They can in some cases lead to an enhancement of the VET signal, as they may feature very narrow and intense peaks within a favorable protein environment (as is the case in Ile325N_3_Phe and Phe325Aha).

Our results show that the different probes can be used interchangeably with peak times of different probes being comparable to within 1.3 ps. As we observe a peak time increase of about 0.6 ps per residue in this effectively linear case, differences can be resolved even for sites i and i+3 . With the comparison of the different labels in dipeptides within different solvents and in the protein environment, we gave insight into the different effects of the environment, which are of higher importance in small systems and in direct contact to the solvent. This knowledge will allow for the design of VET studies in more complex and variable protein environments, paving the way to further elucidate how VET is involved in protein dynamics and function.

## Conflict of interest

The authors declare no conflict of interest.

1

## Supporting information

As a service to our authors and readers, this journal provides supporting information supplied by the authors. Such materials are peer reviewed and may be re‐organized for online delivery, but are not copy‐edited or typeset. Technical support issues arising from supporting information (other than missing files) should be addressed to the authors.

Supporting Information

## Data Availability

The data that support the findings of this study are available from the corresponding author upon reasonable request.
