# Supplementary material for: Versatile Vibrational Energy Sensors for Proteins
Source: Angew Chem Int Ed Engl. 2022 Apr 6;61(21):e202200648. doi: 10.1002/anie.202200648 (PMC9401566; doi:10.1002/anie.202200648)
Supplement: Supplementary file 1 — Supporting Information [file ANIE-61-0-s001.pdf]

Angewandte Chemie
Eine Zeitschrift der Gesellschaft Deutscher Chemiker

Supporting Information

# Versatile Vibrational Energy Sensors for Proteins

*J. G. Löffler, E. Deniz, C. Feid, V. G. Franz, J. Bredenbeck**

## Table of Contents

1. Experimental procedures
    1.1 Sample synthesis
    1.2 Sample preparation
    1.3 UV/VIS and FTIR spectroscopy
    1.4 Transient VIS-pump/IR-probe spectroscopy
    1.5 Data treatment
2. Supporting results

# 1. Experimental procedures

## 1.1 Sample synthesis

**Amino acids and peptides.** The free AzAla and CNTrp were enzymatically synthesized in-house using the strategies described by Watkins et al.[1] and Boville et al.,[2] respectively. The plasmids containing the genes for TmAzul and Tm9D8* were a gift by Frances H. Arnold. The raw AzAla was handed to sb-PEPTIDE (France) for the synthesis of all peptides using solid phase peptide synthesis and an Fmoc approach. The supplier determined purities of 86% for AzAla-$N_3$Ala and >95% for the other peptides by HPLC. All peptides were used without further purification.
**Proteins.** For all expressions of PDZ3, plasmids containing a gene coding for PDZ3 with an N-terminal His-tag before a TEV-cleavage site under the control of a T5 promoter and the lac operator were used. For the incorporation of Aha, the codon at the desired position was substituted with a Met-codon, for the incorporation of SCN with a Cys-codon, and for the substitution with $N_3$Phe and CNPhe with an amber stop-codon, using standard PCR-mutagenesis protocols. The final substitutions were confirmed via Sanger sequencing. Appropriate *E. coli* strains were transformed with these plasmids via electroporation, spread on agar plates, and one single colony was picked for expression. All growth plates and media contained ampicillin for the selection of the PDZ plasmid.
The expression of the wild-type PDZ and the Ile327SCN variant were done in BL21 (DE3) cells in Luria broth (LB) medium. After incubation at 37°C until reaching an $OD_{600}$ of 0.7, the expression was induced by the addition of 1 mM IPTG and continued overnight at room temperature.
For expression of Aha mutants in *E. coli* B834 (DE3) (Novagen), the cells were grown in LB medium overnight at 37°C. The cells were then centrifuged, washed, resuspended, and transferred to M9 minimal media supplemented with 4 g/l glucose, 2 mM $MgSO_4$, 0.1 mM $CaCl_2$, 1 mg/l riboflavin, nicotinamide, pyridoxal hydrochloride, and thiamine, trace elements, and 0.5 mM Aha. After incubation at room temperature and reaching an $OD_{600}$ of 1.5, 0.1 g/l lysine, threonine, and phenylalanine, and 50 mg/l leucin, isoleucine, and valine were added, and the expression was induced by adding 1 mM IPTG, and continued overnight.
The incorporation of $N_3$Phe and CNPhe was done in *E. coli* BL21 Star (DE3) cells (Invitrogen) using an SCS approach with the pUltra-CNF plasmid[3] (Addgene) containing an additional D286R mutation, to increase the suppression efficiency.[4] Growth plates and cultures for this approach were additionally supplemented with streptomycin for the selection of the pUltra plasmid. The culture in terrific broth (TB) medium supplemented with 1 mM of the desired ncAA was induced by the addition of 1 mM IPTG at a cell density of 0.6-1.0 OD, and further incubated overnight at 37°C.
All cells were harvested by centrifugation at 8000 rpm at 4°C and disrupted via ultrasonication. DNA was precipitated using 0.5% w/v streptomycin sulfate. DNA and cell debris were removed by ultracentrifugation at 35000 rpm for 45 min at 4°C.
For the protein purification, the filtered supernatant was applied to a Ni-NTA column, binding the His-tag of the target protein, and eluted using imidazole. After elution, the His-tag was removed by overnight TEV-cleavage, while simultaneously removing the imidazole via dialysis. The dialysate was again applied to the Ni-NTA column, removing the free His-tag and the His-tagged TEV-protease, the target protein remained in the flowthrough. The final purification was achieved by size exclusion chromatography on a SuperDex PG 75 column. The protein was stored at -70°C in 50 mM sodium phosphate buffer pH 6.8 until further use.
The final cyanylation reaction of PDZ Ile327SCN was conducted as described for myoglobin previously[5] with minor adaptations. The procedure is based on the DTNB approach described by Fafarman et al.[6] For the cyanylation, isotope-labelled $K^{13}C^{15}N$ was used, shifting the infrared absorption by about -80 $cm^{-1}$ to 2082 $cm^{-1}$ as compared to $S^{12}C^{14}N$. Throughout the text SCN is used to describe the $S^{13}C^{15}N$, omitting the explicit mention of the isotopes.

## 1.2 Sample preparation

**Dipeptides.** Dipeptide samples for FTIR and VET measurements were prepared by dissolving 1 mg of the respective peptide in either 60 µl of tetrahydrofuran (THF) or dimethyl sulfoxide (DMSO). Aqueous samples were prepared in 20 µl 250 mM NaOH and diluted by addition of 40 µl $H_2O$.
The samples were mounted between two $CaF_2$ windows separated by a 100 µm PTFE spacer, sealed with PTFE paste. The complete cell was moved by a Lissajous scanner within the focal plane during the measurement to continuously refresh the sample.
**Proteins.** For the laser experiments, the protein was thawed on ice and concentrated to an appropriate concentration using centrifugal concentrators with a cut-off of 3 kDa. An AzAla-KQTSV peptide stock solution in sodium phosphate buffer pH 6.8 was prepared and

the final concentration verified using UV/VIS spectroscopy and the absorption at 341 nm. The ligand was added to the protein in ~20% molar excess, resulting in final protein concentrations of 18 mM for I327SCN and ~8-11 mM for the other variants. The same procedure was followed to prepare a sample of wild-type PDZ (without the IR label) at a comparable concentration.
The protein samples were mounted in a sample cell made of two $CaF_2$ windows with a 100 µm PTFE spacer. This cell contained two sample chambers, separated by a barrier of PTFE and sealed with PTFE paste to prevent leakage from one compartment to the other (manuscript accepted[7]). One of these compartments contained the PDZ mutant with the respective probe ncAA and the VET ligand, the other compartment contained the wild-type PDZ sample with the VET ligand. After each scan, the sample compartment was switched to enable quasi-simultaneous measurement of the background signal in the wild-type compartment.
The complete cell was moved by a Lissajous scanner within the focal plane during the measurement to continuously refresh the sample.

## 1.3 UV/VIS and FTIR spectroscopy

Final dipeptide concentrations were determined using the AzAla absorption at 612 nm with an extinction coefficient of 328, 360 and 317 $M^{-1}$ $cm^{-1}$ in DMSO, THF, and $H_2O$ respectively. The extinction coefficients were determined from concentration series of the AzAla-Aha dipeptide. Protein concentrations were determined using the UV absorbance at 280 nm. All UV/VIS spectra were recorded with a Hitachi U-2000 spectrometer against the respective solvent background.
FTIR spectra were recorded on a Bruker Tensor 27 FTIR spectrometer with 60 µm path length for aqueous and DMSO samples and 100 µm path length for samples in THF against the respective solvent background. The FTIR peaks of azide and nitrile vibrations were further isolated by fitting a polynomial to the region around the respective signal, omitting the peak itself.

## 1.4 Transient VIS-pump/IR-probe spectroscopy

A commercial amplified Ti:sapphire laser system (Mai Tai (oscillator), Empower (pump laser), Spitfire ace (amplifier) from Spectra Physics) was used to generate ~100 fs pulses of 800 nm light with a repetition rate of 3 kHz. The output of this laser system was split in two parts to operate two independent home-built optical parametric amplifiers (OPA). Both OPAs use a β-barium-borate (BBO) crystal in two amplification steps. In one OPA, difference frequency generation of signal and idler in a silver thiogallate ($AgGaS_2$) crystal was used to generate probe and reference pulses around 2100 $cm^{-1}$. In the second OPA, the signal was frequency-doubled in another BBO to generate 612 nm pump pulses with 6 µJ pulse energy. The beams were focused into the sample (80 µm and 125 µm FWHM for IR and VIS beam, respectively). Pump and probe beam overlapped, the reference beam passed the sample roughly 2 mm above the other beams and was used to correct for fluctuations in the OPA output. The relative angle between the polarization of pump and probe beam was set to the magic angle for all experiments.
IR pulses were dispersed on a 150 l/mm or 300 l/mm grating in a spectrometer (Triax 180, Jobin Yvon) and detected on a 2x64 pixel MCT array detector (Infrared Systems Development). A chopper wheel in the pump path allowed to measure unpumped/pumped spectra consecutively. The detector readout was linearized, referenced, difference spectra calculated and saved using home-written software (Python 3).
A mechanical delay stage M-406.6PD (Physik Instrumente) was used to vary the delay between the pump and probe pulse, after recording 150000 (for shortest and longest delay time) or 30000 (all other delays) laser shots. All delays were scanned multiple times, and the data were averaged.

## 1.5 Data treatment

The difference signal at -40 ps delay time was subtracted as long-lived background from all other time points. The pump-probe data recorded in THF and DMSO were analyzed without further treatment. The samples in $H_2O$ show an intense background of solvent heating in the probed IR region. To correct for this thermal background, we scaled the difference spectrum of a late delay, i.e., after the decay of the VET-induced signal, to each earlier time point together with an offset parameter, assuming a constant band shape and subtracted it (Figure S1).[8] Due to the large amplitude of the dipeptide signals, the region of the signal was omitted from this fit, leaving the pixels without azide or nitrile signal for the scaling of the $H_2O$ background signal.
The data collected from both compartments of the PDZ sample cells were treated the same way. As the signals from the protein domain are much smaller than those of the dipeptides, we used the split sample cell to correct for minor artifacts, that could not be removed by the scaling of the solvent signal alone. To this end, the difference signal from the wild-type compartment was scaled to the difference signal of the mutant compartment at each delay and subtracted. The presented data are the average of two measurements per PDZ mutant.
At each delay, the absolute values of pixels carrying the VET signal were integrated to yield the VET traces. A biexponential fit to the upper 35% of the traces was applied to determine the peak times. For the protein samples containing $N_3$Phe, two distinct VET features can be observed. The traces and peak times reported here are those determined from the main feature. Due to the low signal-to-noise ratio of the side feature, its traces have not been analyzed in detail, but the dynamics appear to be very similar (Figure S3).
For the comparison of the signal sizes, the difference signals of the dipeptides were normalized to the peptide concentrations. The signals of the proteins were normalized to the protein concentration and to the difference signal of the solvent background at 2212 $cm^{-1}$, which represents a measure for the pump energy deposited in the probed sample region. Therefore, as other dependencies have been removed, the sizes of the VET signals in Figures 3 and 4 reflect the differences of the labels and effects of different environments.

To estimate the VET-induced shift of the azide and nitrile bands, we employed two approaches to fit the time-dependent IR difference spectra: (i) directly using the sample's FTIR spectrum as the band shape of both (negative and positive) signal components, and (ii) fitting a Voigt profile to the FTIR spectrum and use the determined parameters to model the negative feature and a Voigt profile with the same area but variable widths to account for the positive feature. The central wavenumber of the positive feature was shifted at each delay, while the position of the negative feature was kept constant.

Due to the strong dependency between the signal amplitude and the shift, the amplitude of the negative component was fixed to 12% of the FTIR signal. This value was determined through calculations based on previous work of van Wilderen et al.[9] Briefly, the percentage of dipeptide excitation along the gaussian pump beam diameter was determined, under consideration of the local beam intensity. The relative probe intensity along the beam diameter was determined and convoluted with the percentual excitation before integration, resulting in the percentage of molecules that were probed and previously excited within the probed volume.

## 2. Supporting results

**Table S1:** Parameters determined from the FTIR spectra of the various AzAla-ncAA dipeptides in different solvents. FWHM: full width at half maximum; B: integrated extinction coefficient; $\omega_{max}$: wavenumber of the maximum.

| | $H_2O$ | | | DMSO | | | THF | | |
|---|---|---|---|---|---|---|---|---|---|
| | FWHM [$cm^{-1}$] | B [$cm^{-1}$/cm/M] | $\omega_{max}$ [$cm^{-1}$] | FWHM [$cm^{-1}$] | B [$cm^{-1}$/cm/M] | $\omega_{max}$ [$cm^{-1}$] | FWHM [$cm^{-1}$] | B [$cm^{-1}$/cm/M] | $\omega_{max}$ [$cm^{-1}$] |
| **$N_3$Ala** | 18.6 | 14,000 | 2115.7 | 23.9 | 11,700 | 2106 | 24.6 | 19,100 | 2105.7 |
| **Aha** | 31.4 | 15,000 | 2113.1 | 27.4 | 11,600 | 2100 | 27.8 | 23,100 | 2099.7 |
| **$N_3$Phe** | 34.5 | 13,700 | 2026.9 | 23.4 | 12,800 | 2116.5 | 28.7 | 20,000 | 2114.4 |
| **CNPhe** | 10.2 | 2,640 | 2235 | 7.3 | 1,590 | 2226.7 | 5.6 | 1,850 | 2228.4 |
| **CNTrp** | 12.2 | 1,420 | 2219.3 | 8.6 | 1,370 | 2214.4 | 8.0 | 2,140 | 2216.4 |

**Table S2:** Parameters determined from fits to the FTIR spectra of all PDZ mutants. F325$N_3$Phe was fit with Voigt functions, all other fits are Gaussian functions. FWHM: full width at half maximum; B: integrated extinction coefficient; $\omega_{max}$: wavenumber of the maximum.

| | | PDZ I327 | | | PDZ F325 | | |
|---|---|---|---|---|---|---|---|
| | | FWHM [$cm^{-1}$] | B [$cm^{-1}$/cm/M] | $\omega_{max}$ [$cm^{-1}$] | FWHM [$cm^{-1}$] | B [$cm^{-1}$/cm/M] | $\omega_{max}$ [$cm^{-1}$] |
| **Aha (no ligand)** | Peak 1 | 29.6 | 11,680 | 2099.0 | 16.3 | 2,240 | 2088.1 |
| | Peak 2 | | | | 11.5 | 9,660 | 2100.0 |
| **Aha (with ligand)** | Peak 1 | 25.6 | 8,640 | 2097.2 | 16.1 | 2,680 | 2085.9 |
| | Peak 2 | 20.8 | 1,820 | 2110.5 | 9.9 | 8,320 | 2099.4 |
| **$N_3$Phe (no ligand)** | Peak 1 | 20.5 | 7,200 | 2105.2 | 12.9 | 4,140 | 2101.0 |
| | Peak 2 | 29.4 | 10,300 | 2028.3 | 45.0 | 10,100 | 2121.0 |
| **$N_3$Phe (with ligand)** | Peak 1 | 12.7 | 5,300 | 2101.2 | 15.9 | 5,900 | 2100.4 |
| | Peak 2 | 44.8 | 12,900 | 2121.3 | 33.3 | 6,790 | 2125.1 |
| **CNPhe (no ligand)** | | 11.6 | 1,600 | 2236.7 | 5.8 | 812 | 2230.3 |
| **CNPhe (with ligand)** | | 11.4 | 1,350 | 2238.1 | 5.8 | 817 | 2231.5 |
| **SCN (no ligand)** | | 12.6 | 495 | 2083.4 | | | |
| **SCN (with ligand)** | | 7.9 | 270 | 2082.0 | | | |

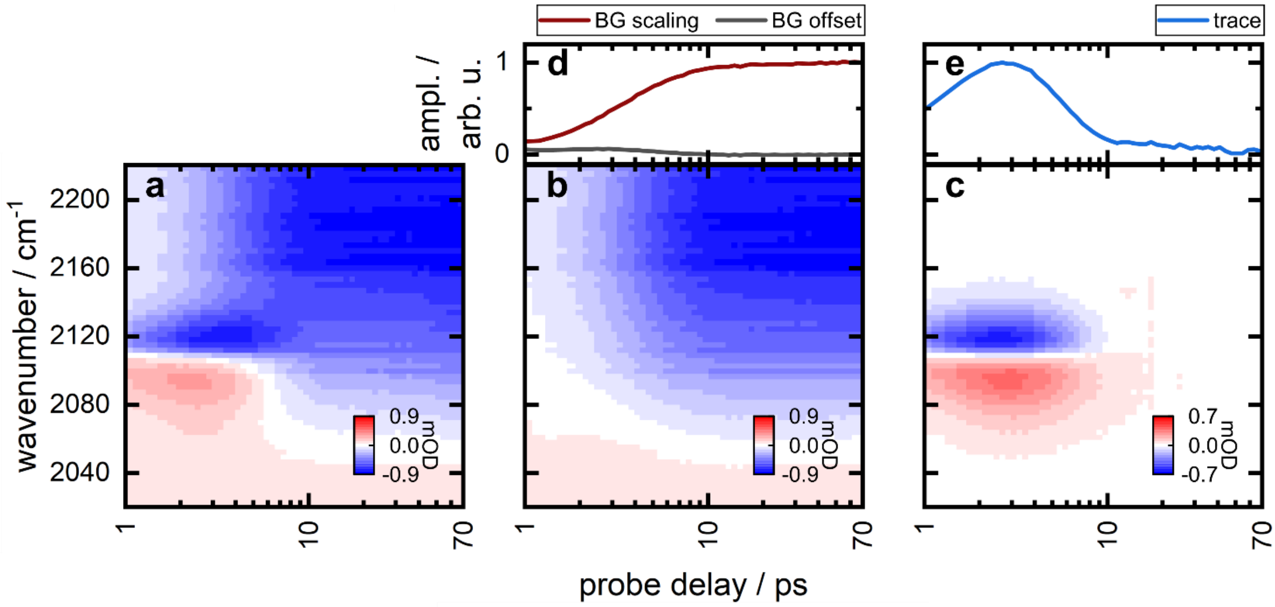


**Figure S1.** (a) Heat map of transient spectra of AzAla-Aha displaying the typical VET signal of the dipeptide overlapping with the thermal response of the water. (b) The background that is subtracted to isolate the VET signal of the IR label. The background results from fitting the difference signal at 73 ps to the earlier delays. (c) Result of the background subtraction. (d) Scaling and offset parameters of the background correction, note the convergence upon thermal equilibration. (e) TRIR trace of the isolated signal.

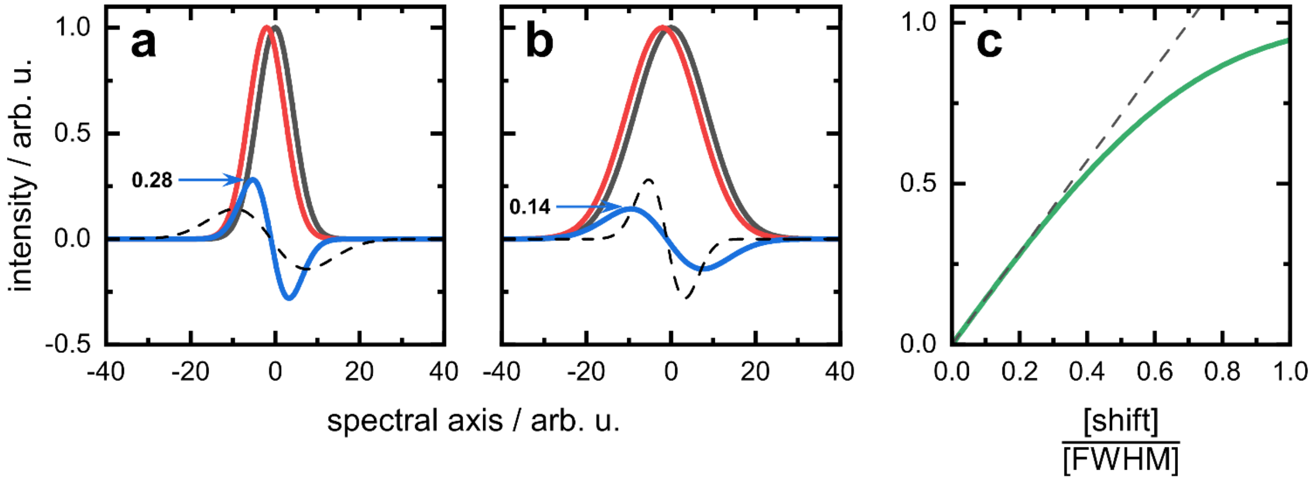


**Figure S2.** The difference signal (blue) of two slightly shifted Gaussian functions (gray and red) is inversely proportional to their FWHM, as shown for a shift of 2 units and FWHM of 10 and 20 units in panels a and b, respectively. The signal of the other panel is shown for comparability (black dashed line). (c) Generally, the signal amplitude (green line) is proportional to the relative shift (quotient of shift and FWHM) for small relative shifts. For larger shifts the amplitude asymptotically approaches 1 (complete separation of the peaks). Lorentzian functions show the same behavior with slightly faster deviation from linearity.

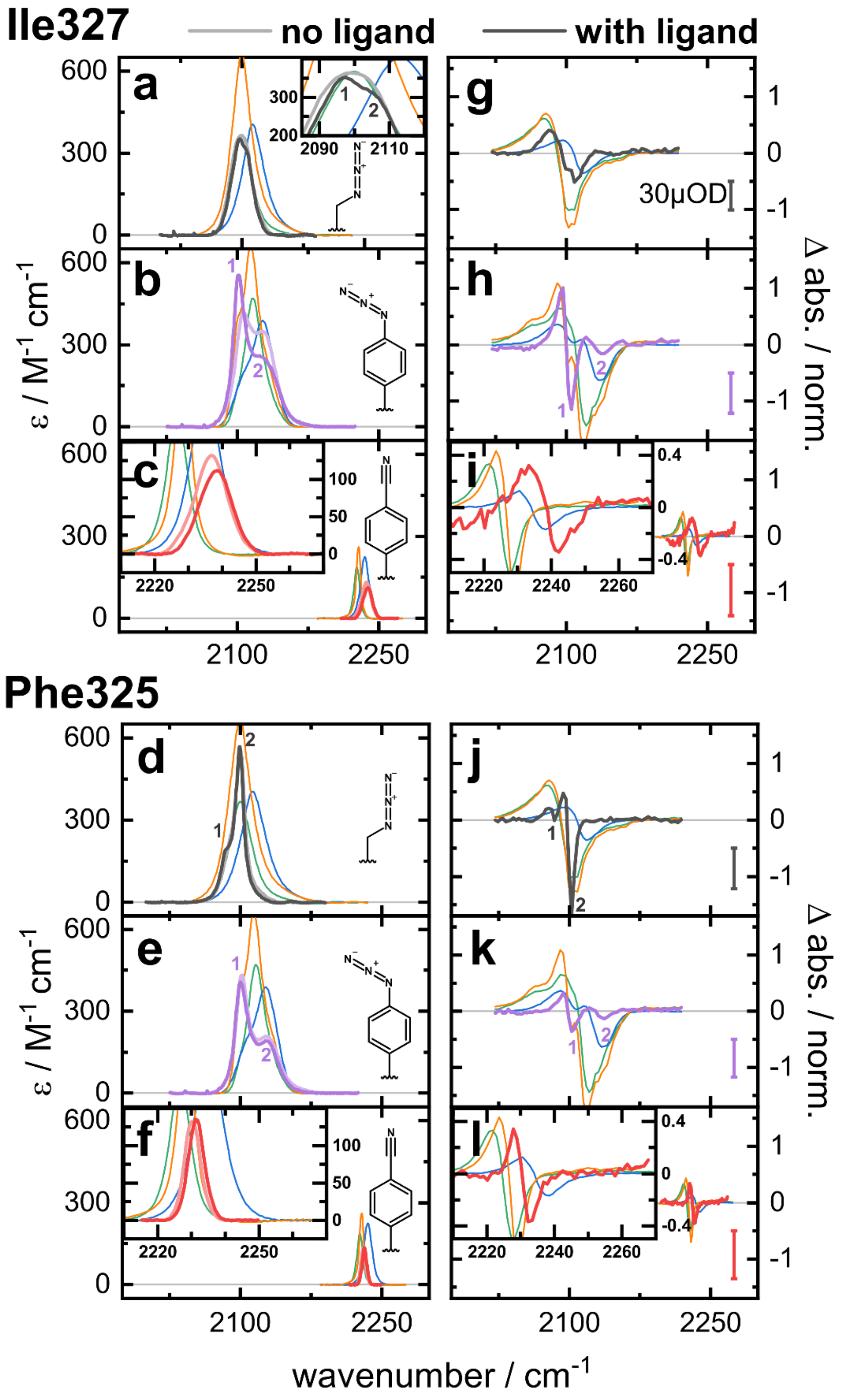


**Figure S3.** (a-f) Extinction coefficients of PDZ variants with Aha (gray), $N_3$Phe (purple), and CNPhe (red), as indicated by the structures. Thin lines represent the extinction coefficients of the dipeptides containing the respective IR probe in the different solvents $H_2O$ (blue), DMSO (green), and THF (orange). Each panel shows the extinction coefficient without the ligand (light colors) and with the ligand (intense colors). (g-l) Difference spectra of the VET experiments at the time of maximum signal intensity of the PDZ mutants (same colors as in the FTIR panels). Thin lines represent the VET signals of the dipeptides containing the indicated IR probe in the different solvents (same colors as in the FTIR panels). The upper panels show the results of PDZ with labels inserted at Ile327, the lower panels show data for PDZ labels inserted at Phe325 instead. The PDZ VET signal intensity is scaled to the protein concentration and the difference signal of water at 2212 $cm^{-1}$ for each experiment so that signal amplitudes are comparable. The dipeptide signals have been scaled for better comparability. Colored bars indicate 30 μOD difference signal in the PDZ VET experiments. Small numbers in the FTIR and VET spectra of Aha and $N_3$Phe containing mutants indicate the presence of multiple subbands.

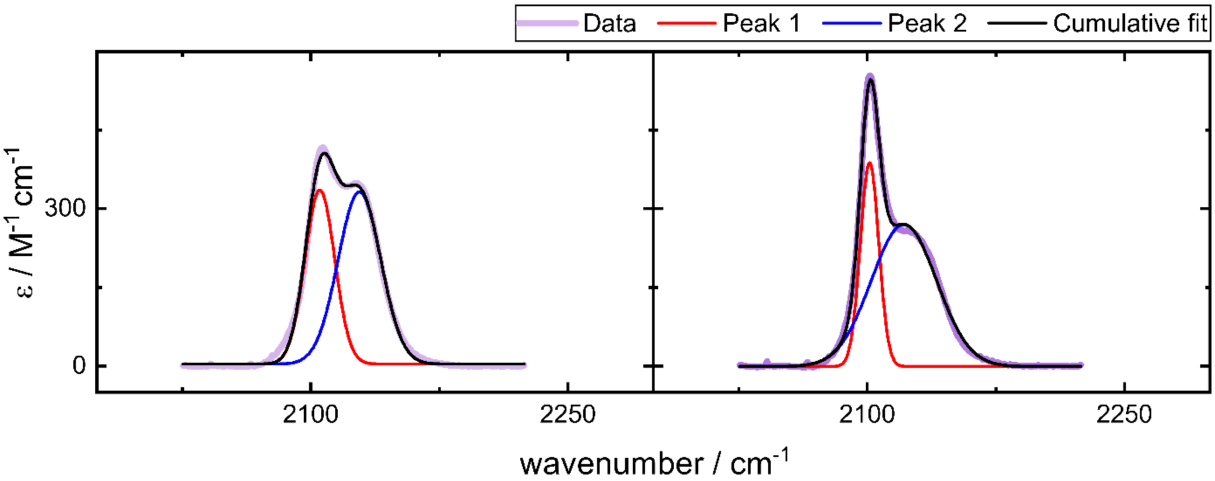


**Figure S4.** FTIR extinction coefficients and fits of PDZ Ile327pN$_3$Phe without (left) and with Azu-KQTSV peptide (right). Each panel shows the extinction coefficient (purple), the fits of two individual Gaussian peaks (red and blue), and the cumulative fit (black). The fitted parameters of all peaks are shown in Table S2.

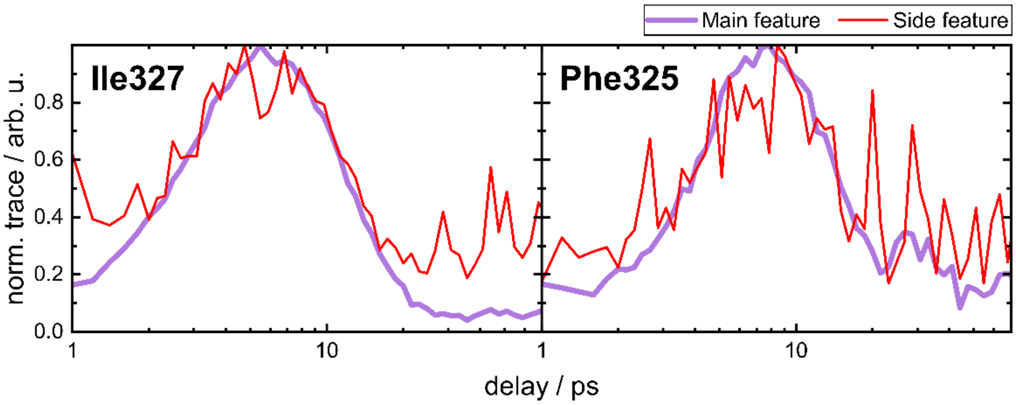


**Figure S5.** Comparison of the dynamics of the two features in the VET signal of PDZ N$_3$Phe mutants. The general dynamics of both features appear similar. The signal-to-noise ratio of the side feature is significantly lower than that of main feature, due to the lower amplitude.

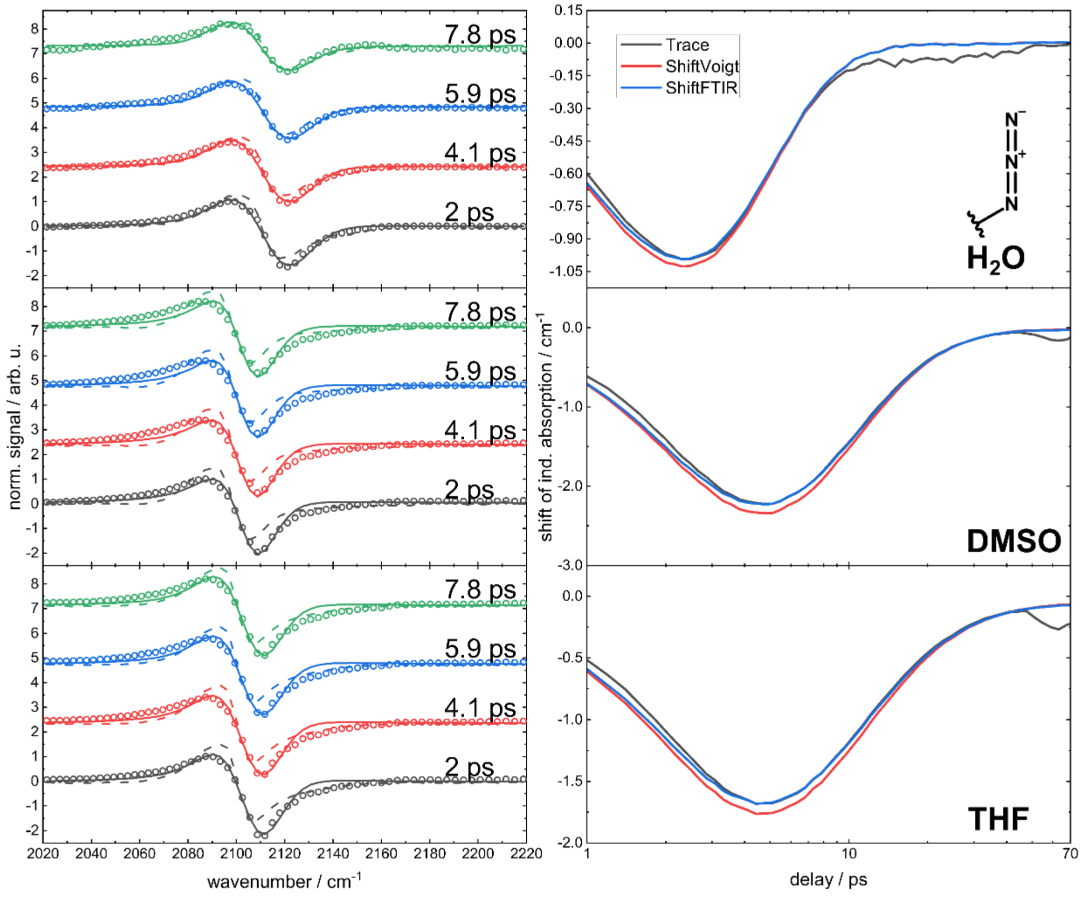


**Figure S6.** Extraction of the shift of the VET-induced absorption relative to the bleach in AzAla-N$_3$Ala in $H_2O$, DMSO and THF (top to bottom). (left) TRIR data (circles) at four exemplary time points (as indicated). Fit using the FTIR spectrum with an amplitude of -12% for the bleach and a shifted FTIR spectrum with an amplitude of +12% for the VET-induced absorption (dashed line) and fit using a Voigt band shape fitted to the FTIR spectrum with an amplitude of -12% modelling the bleach and a Voigt band shape with variable widths but the same area as the band representing the bleach (solid line). For clarity the spectra and fits have been scaled to the maximum of the TRIR data at each time point and offset. (right) Shift as determined for each time point by the fit with both different methods, shifted FTIR spectrum (blue) and shifted Voigt (red). For comparison with the dynamics determined from the TRIR data itself, the TRIR trace was scaled to the shift of the FTIR fit (gray).

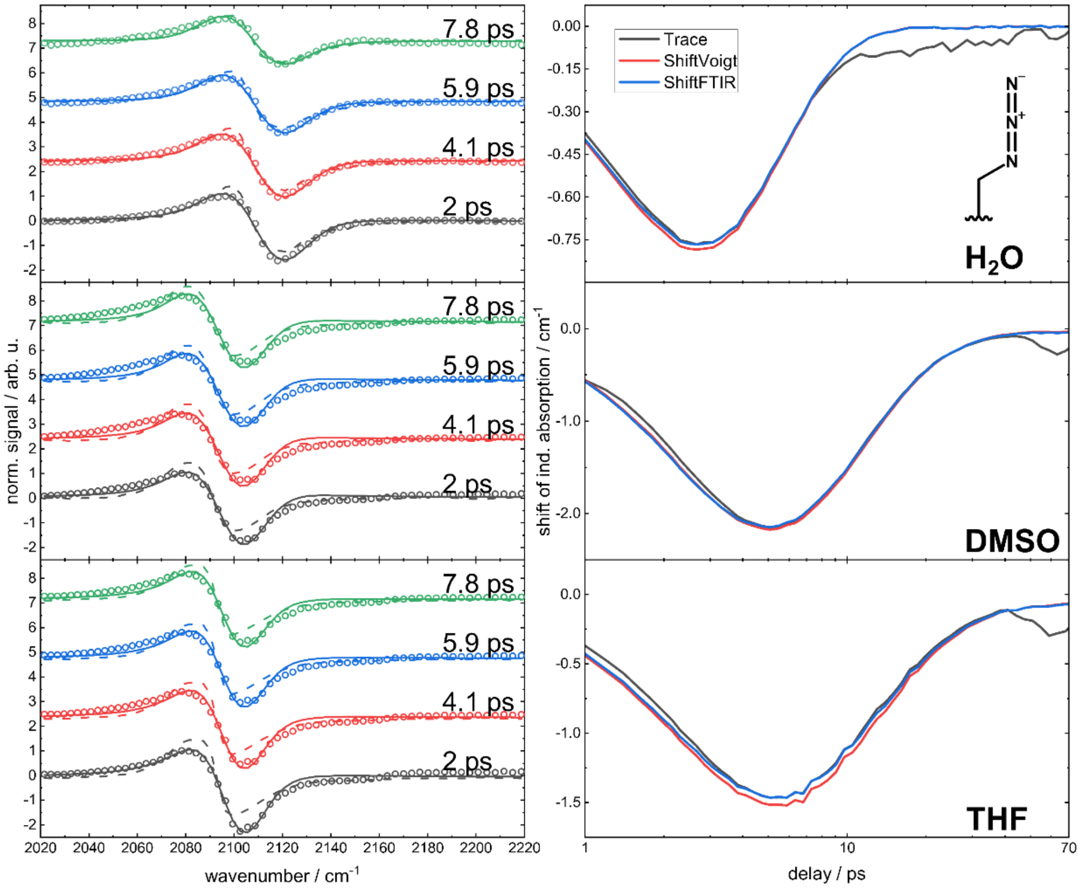


**Figure S7.** Extraction of the shift of the VET-induced absorption relative to the bleach in AzAla-Aha in $H_2O$, DMSO and THF (top to bottom). (left) TRIR data (circles) at four exemplary time points (as indicated). Fit using the FTIR spectrum with an amplitude of -12% for the bleach and a shifted FTIR spectrum with an amplitude of +12% for the VET-induced absorption (dashed line) and fit using a Voigt band shape fitted to the FTIR spectrum with an amplitude of -12% modelling the bleach and a Voigt band shape with variable widths but the same area as the band representing the bleach (solid line). For clarity the spectra and fits have been scaled to the maximum of the TRIR data at each time point and offset. (right) Shift as determined for each time point by the fit with both different methods, shifted FTIR spectrum (blue) and shifted Voigt (red). For comparison with the dynamics determined from the TRIR data itself, the TRIR trace was scaled to the shift of the FTIR fit (gray).

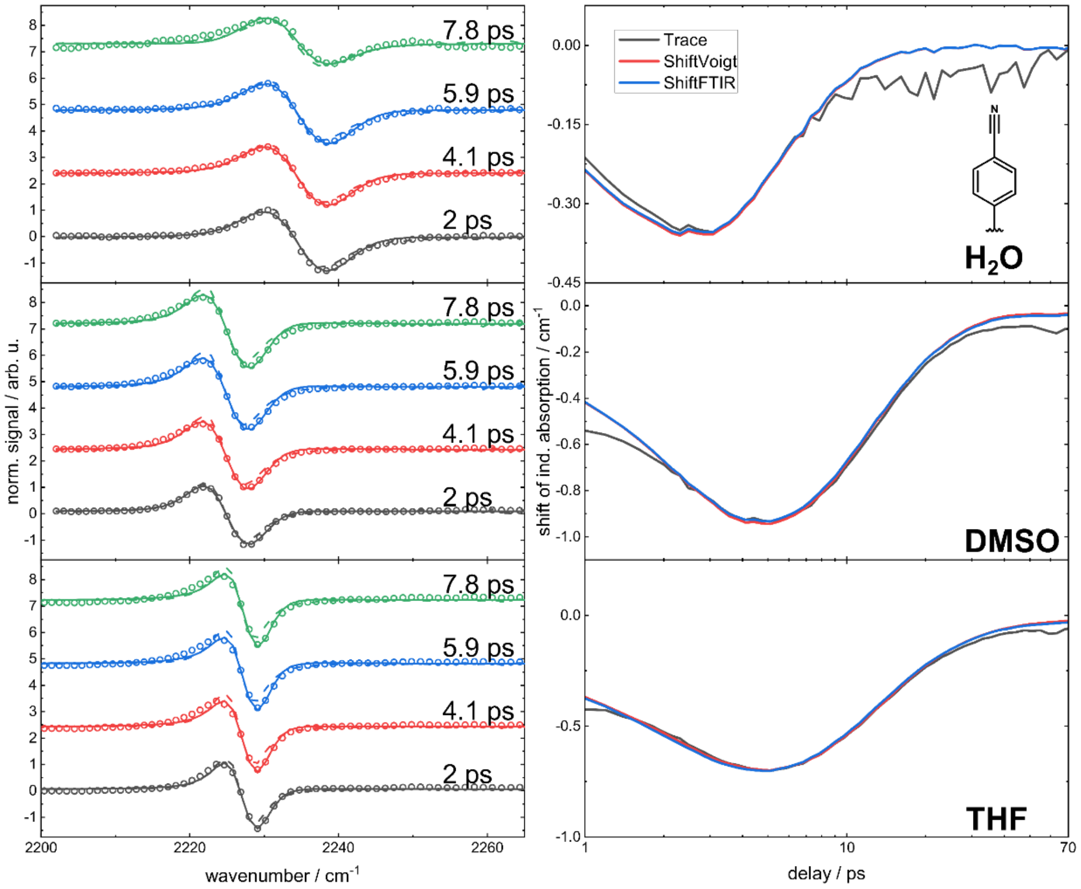


**Figure S8.** Extraction of the shift of the VET-induced absorption relative to the bleach in AzAla-CNPhe in $H_2O$, DMSO and THF (top to bottom). (left) TRIR data (circles) at four exemplary time points (as indicated). Fit using the FTIR spectrum with an amplitude of -12% for the bleach and a shifted FTIR spectrum with an amplitude of +12% for the VET-induced absorption (dashed line) and fit using a Voigt band shape fitted to the FTIR spectrum with an amplitude of -12% modelling the bleach and a Voigt band shape with variable widths but the same area as the band representing the bleach (solid line). For clarity the spectra and fits have been scaled to the maximum of the TRIR data at each time point and offset. (right) Shift as determined for each time point by the fit with both different methods, shifted FTIR spectrum (blue) and shifted Voigt (red). For comparison with the dynamics determined from the TRIR data itself, the TRIR trace was scaled to the shift of the FTIR fit (gray).

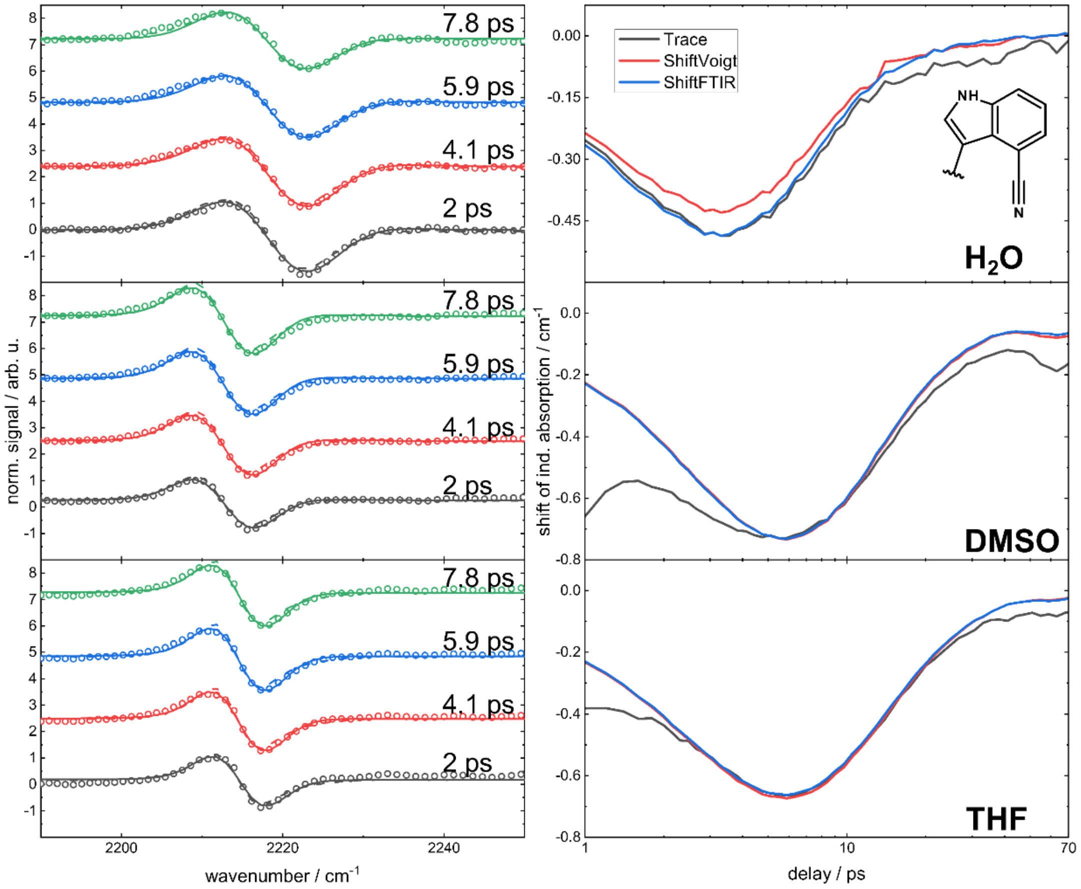


**Figure S9.** Extraction of the shift of the VET-induced absorption relative to the bleach in AzAla-CNTrp in $H_2O$, DMSO and THF (top to bottom). (left) TRIR data (circles) at four exemplary time points (as indicated). Fit using the FTIR spectrum with an amplitude of -12% for the bleach and a shifted FTIR spectrum with an amplitude of +12% for the VET-induced absorption (dashed line) and fit using a Voigt band shape fitted to the FTIR spectrum with an amplitude of -12% modelling the bleach and a Voigt band shape with variable widths but the same area as the band representing the bleach (solid line). For clarity the spectra and fits have been scaled to the maximum of the TRIR data at each time point and offset. (right) Shift as determined for each time point by the fit with both different methods, shifted FTIR spectrum (blue) and shifted Voigt (red). For comparison with the dynamics determined from the TRIR data itself, the TRIR trace was scaled to the shift of the FTIR fit (gray).

## References

[1] E. J. Watkins, P. J. Almhjell, F. H. Arnold, *Chembiochem : a European journal of chemical biology* **2020**, *21*, 80.
[2] C. E. Boville, D. K. Romney, P. J. Almhjell, M. Sieben, F. H. Arnold, *J. Org. Chem.* **2018**, *83*, 7447.
[3] a) D. D. Young, T. S. Young, M. Jahnz, I. Ahmad, G. Spraggon, P. G. Schultz, *Biochemistry* **2011**, *50*, 1894; b) K. C. Schultz, L. Supekova, Y. Ryu, J. Xie, R. Perera, P. G. Schultz, *J. Am. Chem. Soc.* **2006**, *128*, 13984.
[4] T. Kobayashi, O. Nureki, R. Ishitani, A. Yaremchuk, M. Tukalo, S. Cusack, K. Sakamoto, S. Yokoyama, *Nat. Struct. Biol.* **2003**, *10*, 425.
[5] L. J. G. W. van Wilderen, D. Kern-Michler, H. M. Müller-Werkmeister, J. Bredenbeck, *Phys. Chem. Chem. Phys.* **2014**, *16*, 19643.
[6] A. T. Fafarman, L. J. Webb, J. I. Chuang, S. G. Boxer, *J. Am. Chem. Soc.* **2006**, *128*, 13356.
[7] E. Deniz, J. G. Löffler, A. Kondratiev, A. R. Thun, Y. Shen, G. Wille, J. Bredenbeck, *Rev. Sci. Instrum.* **2022**, accepted DOI: 10.1063/5.0079958.
[8] a) T. Baumann, M. Hauf, F. Schildhauer, K. B. Eberl, P. M. Durkin, E. Deniz, J. G. Löffler, C. G. Acevedo-Rocha, J. Jaric, B. M. Martins, H. Dobbek, J. Bredenbeck, N. Budisa, *Angew. Chem. Int. Ed. Engl.* **2019**, *58*, 2899; b) E. Deniz, L. Valiño-Borau, J. G. Löffler, K. B. Eberl, A. Gulzar, S. Wolf, P. M. Durkin, R. Kaml, N. Budisa, G. Stock, J. Bredenbeck, *Nat. Commun.* **2021**, *12*, 3284.
[9] L. J. G. W. van Wilderen, C. N. Lincoln, J. J. van Thor, *PloS one* **2011**, *6*, e17373.

## Author Contributions

JGL and ED: performed the investigation, data curation, data analysis and data visualization, wrote the original draft, reviewed & edited the final manuscript. CF and VGF: prepared resources (AzAla and CNTrp and IR-labelled proteins). JB: conceptualization, funding acquisition, project administration and supervision, reviewed & edited the final manuscript.
